# Escaping Constraints to Innovate: Maternal Neofunctionalization in a *HoxB4* Duplicate

**DOI:** 10.1002/jezb.70012

**Published:** 2026-01-12

**Authors:** Júlia de Lima Carvalho, José Caetano Silva‐Filho, Janaina Lima de Oliveira

**Affiliations:** ^1^ Instituto de Biologia Universidade Federal da Bahia Salvador Bahia Brazil; ^2^ UNIESP Centro Universitário Cabedelo Paraíba Brazil; ^3^ Afya Faculdade de Ciências Médicas da Paraíba Cabedelo Paraíba Brazil

**Keywords:** Evo‐devo, intrinsically disordered regions, maternal expression, positive selection, transcription factor evolution, *Xenopus laevis*

## Abstract

Transcription factors are typically thought to play a limited role in developmental evolution due to their high pleiotropic nature. However, such constraints may be relaxed following gene duplication or when proteins are organized into structural and functional modules, opening avenues for evolutionary innovation. Here, we integrate expression and genomic data to investigate the evolutionary dynamics of *Hox* gene duplicates in the allotetraploid frog *Xenopus laevis*. Despite overall conservation across the *Hox* clusters, we find that *HoxB4L* has acquired expression during maternally regulated stages and is evolving under positive selection. Protein‐level changes include the number, length, and sequence of functionally important protein regions. Our results indicate that *HoxB4L* has escaped ancestral constraints and is undergoing maternal neofunctionalization as a result of cis‐regulatory divergence and structural protein modifications. These findings illustrate how transcription factors can overcome developmental constraints and contribute to novel functions during early development.

## Graphical abstract

Whole‐genome duplication in *Xenopus laevis* generated duplicated *Hox* genes that are largely constrained in sequence and developmental expression. However, *HoxB4L* uniquely acquired maternal expression through cis‐regulatory and protein structural divergence, illustrating how gene duplication enables transcription factors to escape pleiotropic constraints and evolve novel roles in early development.

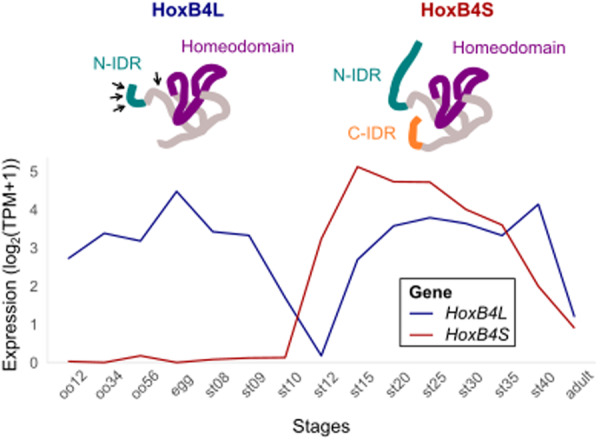

## 1 Introduction

Embryonic development is a finely orchestrated process in which slight changes may be amplified due to the highly pleiotropic nature of developmental genes. Nonetheless, developmental programmes are subject to evolutionary change, sometimes resulting in significant morphological innovations (Carroll et al. 2004). The understanding of how the molecular architecture of development evolves, and how it relates to morphological diversification, has been further challenged by the discovery of toolkit genes (Duboule and Dollé 1989; Graham et al. 1989): how can a conserved developmental genetic toolkit give rise to the vast diversity of life forms observed? (Carroll et al. 2004; Cañestro et al. 2007). A similar paradox was posed by King and Wilson's observation that the high degree of DNA sequence similarity between humans and chimps cannot fully account for their substantial organismal (anatomical, physiological, behavioural, and ecological) differences. These, they proposed, must largely stem from regulatory changes (King and Wilson 1975). Building upon these (and other (Carroll et al. 2004)) insights, as well as Jacob's concept of evolution by tinkering (Jacob 1977), Evolutionary Developmental Biology (Evo‐Devo) has recognised regulatory evolution as a major driver of evolutionary innovation (Carroll et al. 2004; Carroll 2005; Cañestro et al. 2007). The rationale is that, while structural changes in proteins tend to have widespread pleiotropic effects, modifications in cis‐regulatory modules affect gene expression only in specific developmental contexts. This helps explain how developmental and morphological evolution can occur despite the pervasive structural conservation of toolkit genes.

However, the contribution of protein‐mediated developmental evolution has been underestimated (Hoekstra and Coyne 2007; Lynch and Wagner 2008; Wagner and Lynch 2008). Structural evolution of highly pleiotropic master regulators of development is well documented (see (Lynch and Wagner 2008) for a review) and can be understood in light of two main concepts. First, there is a common confusion between the pleiotropic roles of genes and the pleiotropic effects of mutations (Stern 2000). Although many genes participate in multiple developmental processes, individual mutations need not have functional effects in every tissue in which the gene is expressed. Indeed, the same logic of modular evolution can be applied to protein sequence and structure, since specific domains or regions may mediate protein‐protein interactions in some tissues but not in others (Hoekstra and Coyne 2007; Lynch and Wagner 2008; Wagner and Lynch 2008; Cheatle Jarvela et al. 2014). Second, the strong constraints imposed by pleiotropy can be relaxed following gene duplication, owing to functional redundancy. This generates genetic variation upon which selection may act to create novel functions (neofunctionalization) (Ohno 1970; Lynch and Conery 2000). Importantly, regulatory and protein‐mediated developmental evolution are not mutually exclusive. Rather, they can interact to create molecular innovations after duplication events (Hoekstra and Coyne 2007), with natural selection shaping coding sequences to create new functions, while modifying regulatory regions to recruit genes to novel expression domains.

*Hox* genes are fundamental components of the bilaterian developmental toolkit, involved in anteroposterior (AP) patterning of all three germ layers (ectoderm, mesoderm and endoderm). This is carried out by a finely tuned ‘*Hox* code’, in which combinations of molecular products from different *Hox* genes are deployed along the AP axis to specify segment identity (Pearson et al. 2005). *Hox* genes encode transcription factors that bind regulatory regions of target genes through the homeodomain, which is conserved among *Hox* paralogues and orthologues (Pick and Au 2025). These proteins also contain intrinsically disordered regions (IDRs), which modulate DNA‐binding affinity and are thought to contribute to context‐specific functions (Bondos and Hsiao 2012; Salomone et al. 2024). The *Hox* gene family is organized into a single cluster in invertebrates (Lewis 1978) and basal chordates (Garcia‐Fernàndez and Holland 1994), but into four clusters (A−D) in vertebrates. This expansion resulted from two rounds of whole‐genome duplication (WGD) (Escriva et al. 2002), a major event in vertebrate evolution. The expansion and diversification of the *Hox* repertoire enabled increased anatomical complexity (Carroll 1995; Singh and Krumlauf 2022), while changes in their regulatory dynamics contributed to the emergence of the vast diversity of body forms (Carroll 1995).

Additional rounds of WGD occurred in several vertebrate lineages, including teleost fish (Glasauer and Neuhauss 2014) and the *Xenopus* group of African clawed frogs (Evans 2008). *Xenopus laevis* has received special attention for studies on polyploid genome evolution, mainly because it is a model organism for developmental studies (Fainsod and Moody 2022). *X. laevis* arose from the hybridization of two ancient diploid species, followed by a WGD event nearly 17–18 Mya. As a result, it is an allotetraploid with two subgenomes, named *L* and *S*. Duplicate gene copies inherited from each progenitor species – i.e., homeologue pairs – are named likewise (e.g., *HoxA1L* and *HoxA1S*). The *X. laevis* genome retains ~56% of duplicated genes, but this rises to 98% within the *Hox* clusters (Session et al. 2016; Kondo et al. 2017). Duplicates of nearly all 38 amphibian *Hox* genes have been retained, except for *HoxB2L*, which has become a pseudogene (Session et al. 2016; Kondo et al. 2017). It has been hypothesized that this outstanding retention rate may reflect selective pressure to maintain stoichiometric balance in gene expression, or that retained copies have undergone sub‐ or neofunctionalization (Session et al. 2016).

Canonical *Hox* roles are mediated by proteins expressed from the embryo genome (zygotic expression), but transcriptome analyses have revealed that *HoxB4L* is also maternally expressed in *X. laevis*, suggesting maternal neofunctionalization (Session et al. 2016). Maternal transcripts support development during oogenesis until the beginning of zygotic transcription, which occurs approximately at stages 8‐9 in this species (Sheets et al. 2017). During this prezygotic interval, the embryo relies exclusively on maternal RNAs for cell cycle progression and the establishment of initial body asymmetries (e.g., animal‐vegetal axis and posterior dorsoventral axis (Sheets et al. 2017). Maternal expression of *Hox* genes has also been documented in annelids (Maslakov et al. 2021), myriapods (Chipman et al. 2014), mammals (Paul et al. 2011), fishes (Jakovlić and Wang 2016), ants (Rafiqi et al. 2020), butterflies (Ferguson et al. 2014), and crustaceans (Jaramillo et al. 2022). These genes have been hypothesized to participate in the control of oocyte maturation, the maternal‐to‐zygotic transition, or the first steps of embryo differentiation (Paul et al. 2011), epigenetic adjustment of the zygotic genome (Maslakov et al. 2021), or patterning roles in the unfertilized oocyte (Ferguson et al. 2014).

Sequence analyses have shown that six *Hox* genes, including *HoxB4L*, are evolving rapidly (Kondo et al. 2017), although it remains unclear whether this reflects relaxed or positive selection. These scenarios have opposing evolutionary consequences: relaxed selection may lead to pseudogenization and gene loss, whereas positive selection can drive neofunctionalization, promoting molecular innovation and increasing the chances of gene retention (Lynch and Conery 2000). Here, we integrate gene expression and sequence data to investigate signatures of regulatory and protein‐mediated developmental evolution in *Hox* homeologue pairs of *X. laevis*. Our analyses reveal a general pattern of conservation between duplicates, consistent with selection to maintain gene dosage balance. However, *HoxB4L* exhibits a divergent expression profile and accelerated evolutionary rates in putative cis‐regulatory regions. Its coding sequence is also rapidly evolving and has accumulated at least four adaptive non‐synonymous substitutions, three of which are located within an IDR. In addition to these substitutions, the HoxB4L protein shows changes in IDR number and length, resulting in three‐dimensional structure adaptations that may enhance its interactions with DNA regulatory elements. Taken together, our findings support a process of neofunctionalization in *HoxB4L*, associated with a novel role during maternally regulated stages. They further highlight the interplay between regulatory and protein‐mediated developmental evolution in a key toolkit gene. This contributes fundamentally to a broader understanding of how the molecular architecture of development evolves, with potential implications for morphological diversification.

## 2 Results

### 2.1 Expression Patterns are Conserved between *Hox* Homeologues, Except for the *HoxB4* Pair

**Figure 1:**
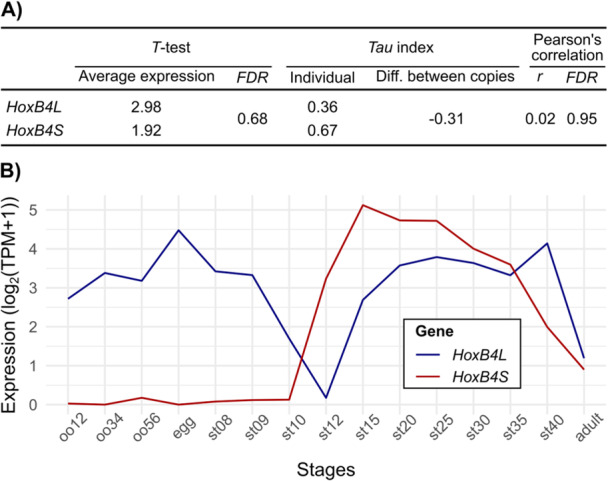
Expression patterns of *HoxB4L* and *HoxB4S* homeologues. (A) Comparison of average expression levels between homeologues was performed using two‐tailed *T*‐tests. Expression specificity was assessed via the *Tau* index (calculated for each copy and its difference), and overall expression profiles were compared using Pearson's correlation coefficients. (B) Temporal expression of *HoxB4L* and *HoxB4S* across developmental and adult stages of *Xenopus laevis*.

Following duplication, gene copies are initially redundant, displaying similar expression levels and profiles. Over time, however, these characteristics may diverge under evolutionary pressures, altering the degree to which expression patterns are retained between gene copies. To investigate how the expression of *Hox* homeologue pairs has evolved since the WGD, we analysed publicly available transcriptomic data from developmental and adult stages (Session et al. 2016). After excluding homeologue pairs in which one copy was a pseudogene or showed no detectable expression, 34 paired copies were compared for average expression levels and overall expression patterns (Table S1). Our results indicate that *Hox* gene homeologues from the *L* and *S* subgenomes generally exhibit highly similar average expression levels (two‐tailed *T*‐tests, *FDR* > 0.05 for all comparisons) and strong positive correlations in expression profiles (median Pearson's correlation *r* = 0.97). A notable exception is the *HoxB4* pair, whose expression profiles are uncorrelated, indicating a divergent pattern (Figure 1A). In particular, *HoxB4L* has the lowest *Tau* specificity index (valued between 0 for housekeeping genes and 1 for tissue‐specific genes) among all *Hox* genes – including its *S* counterpart. Since *HoxB4S* retains a *Tau* value closer to the cluster average (mean *Tau*_*Hox*_ = 0.74), the low *Tau* of *HoxB4L* points to an expression gain in additional developmental contexts. Closer inspection reveals that *HoxB4L* is also expressed in maternally regulated stages (Figure 1B), consistent with previous findings (Session et al. 2016; Kondo et al. 2017).

To determine whether this maternal expression reflects a gain by *HoxB4L* or a loss by *HoxB4S*, we incorporated temporal expression data from *Xenopus tropicalis* (Owens et al. 2016), which provides high‐resolution sampling from eggs (time 0) through the first 66 h of development. Analysis of maternally regulated stages (NF 1 through NF 8‐9; (Sheets et al. 2017)) revealed no detectable *HoxB4* expression in *X. tropicalis*, supporting the interpretation that maternal expression is a derived feature of *HoxB4L* in *X. laevis*. Taken together, these results indicate that *Hox* homeologue expression patterns have remained largely conserved since WGD, with *HoxB4L* representing a clear exception likely co‐opted into maternal expression programmes.

### 2.2 Putative *HoxB4* Cis‐Regulatory Regions Evolve Faster in the *L* Cluster

**Figure 2:**
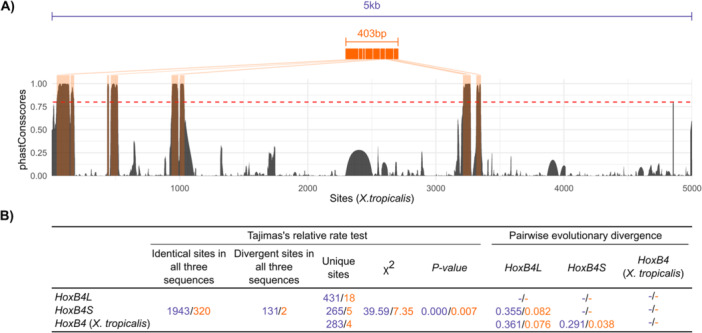
Evolutionary analyses of putative cis‐regulatory regions at the HoxB4L/S loci. (A) Full 5 kb upstream regions of *X. laevis HoxB4L*/*HoxB4S* and *X. tropicalis HoxB4* (purple), with conserved regions (orange) identified based on phastCons scores > 0.8 (red dashed line). Coordinates and sequence length of *X. tropicalis* were used as a reference. (B) Tajima's relative rate tests and pairwise estimates of sequence divergence for the full 5 kb upstream region (purple) and for the concatenated sequence of the eight highly conserved regions across amphibian genomes (orange).

Changes in expression patterns are often driven by modifications in cis‐regulatory regions located near the gene. Consequently, genes co‐opted into new expression contexts are expected to accumulate more differences in these regions, potentially reflected in elevated evolutionary rates. To test whether this applies to *HoxB4L*, we analysed the 5 kb region upstream of the *HoxB4* coding sequences (Figure 2A). Using *X. tropicalis* orthologue as an outgroup, we performed a Tajima's relative rate test (Tajima 1993) and detected a significant excess of substitutions near *HoxB4L*, relative to *HoxB4S* (Figure 2B). Estimates of pairwise distances (Tamura et al. 2004) further support this result (Figure 2B), indicating that the upstream region of *HoxB4L* is more divergent than both the homeologous region in the *S* chromosome and the orthologous region in *X. tropicalis*. To identify candidate regulatory elements within this region, we examined sequence conservation using genomic data from *X. tropicalis* and four additional amphibian species. This analysis revealed eight highly conserved non‐coding regions, which likely correspond to functional regulatory motifs (Figure 2C). When these regions were concatenated and reanalysed, we again found higher evolutionary rates and greater pairwise divergence in those upstream *HoxB4L* compared to *HoxB4S* (Figure 2B). These modifications in cis‐regulatory elements upstream of *HoxB4L* may have enabled its recruitment into maternal expression programmes, leading to its distinct expression profile.

### 2.3 *HoxB4L* has evolved by positive selection

To assess how the coding sequences of *Hox* homeologues have evolved since duplication, we conducted a series of molecular evolution analyses. After excluding homeologue pairs with signs of pseudogenization or substitution saturation (Table S2), we retained 36 *Hox* gene pairs for analysis. For most genes, *L* and *S* copies exhibit similar substitution rates. However, four genes – *HoxB3*, *HoxB4*, *HoxB5* and *HoxB6* – show a significant excess of unique substitutions in their *L* copies (Tajima's relative rate test: *HoxB3L*_*Unique Differences (UD)*_ = 46, *HoxB3S*_*UD*_ = 16, *FDR* = 0.002; *HoxB4L*_*UD*_ = 41, *HoxB4S*_*UD*_ = 14, *FDR* = 0.002; *HoxB5L*_*UD*_ = 55, *HoxB5S*_*UD*_ = 15, *FDR* = 0.000; *HoxB6L*_*UD*_ = 54, *HoxB6S*_*UD*_ = 7, *FDR* = 0.000) (Table S3). These results suggest that the *L* copies of these genes have undergone accelerated coding sequence evolution. Such acceleration may result either from relaxed purifying selection – allowing an accumulation of slightly deleterious mutations – or from positive selection favouring adaptive changes during neofunctionalization. To distinguish between these two scenarios, we used orthologous sequences from five additional amphibian species to identify signatures of selection in each *X. laevis Hox* homeologue.

We first tested for shifts in the stringency of natural selection by performing an analysis that estimates the selection intensity parameter *k* (Wertheim et al. 2015). Values of *k* significantly greater than 1 indicate intensified purifying selection, whereas values less than 1 indicate relaxation. Our analyses detected significant relaxation of selection for *HoxA9S* (*k* = 0.0; *FDR* = 0.0) and *HoxB6L* (*k* = 0.4; *FDR* = 0.0) (Table S4). These results suggest that the high substitution rate observed for *HoxB6L* (Table S3) may reflect a pseudogenization process under weak purifying selection. Although *HoxA9S* has also experienced relaxed constraints, its substitution rate remains similar to that of *Hox9L* (Table S3), possibly indicating that relaxation is recent or of limited magnitude.

We next applied two complementary branch‐site models to test for positive selection on individual branches of the *Hox* gene phylogenies, focusing separately on the *L* and *S* homeologues. These tests estimate the ratio of nonsynonymous to synonymous substitutions (*dN*/*dS* or *ω*), where values of *ω* > 1 indicate positive selection, *ω* ≈ 1 suggests neutral evolution, and *ω* < 1 reflects purifying selection. Both methods identified signatures of positive selection in *HoxB4L* (EasyCodeML: *LRT* = 0.0001; *FDR* = 0.00; aBSREL: *LRT* = 19.3895; *FDR* = 0.01; Table S4). A Bayes Empirical Bayes (BEB) analysis identified four sites under strong positive selection (*HoxB4L* amino acids G32, S34, F35, and T76) with posterior probabilities exceeding 0.99 (Table S4). These results strongly support the hypothesis that *HoxB4L* is undergoing neofunctionalization under positive selection, consistent with its elevated rate of protein evolution.

### 2.4 HoxB4L Exhibits Significant Alterations in Functionally Important Protein Regions

**Figure 3:**
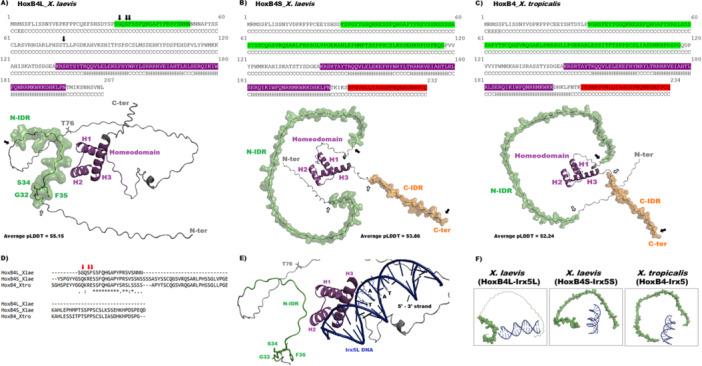
*In silico* analyses of HoxB4 proteins. (A–C) Primary structures and AlphaFold‐predicted 3D models of *X. laevis* HoxB4L (A), HoxB4S (B), and *X. tropicalis* HoxB4 (C) (Abramson et al. 2024). Colours indicate N‐ and C‐terminal intrinsically disordered regions (IDRs) and the homeodomain. Letters below each residue denote secondary structure predicted by PROTEUS2 (Montgomerie et al. 2008) (C = coil; E = β‐strand; H = helix). In (A), arrows highlight the four amino acid residues under positive selection. In the structural models, H1‐H3 refer to homeodomain helices; arrows indicate the first (white) and last (black) residues of each IDR. Average pLDDT scores are shown. (D) Alignment of the N‐IDR region, showing high conservation scores for the 22‐residue segment corresponding to HoxB4L, except for the three positively selected sites indicated by red arrows. Symbols denote residue conservation: (*) identical; (:) conservative substitution; (.) semi‐conservative substitution; () non‐conservative substitution. (E) Predicted HoxB4L‐Irx5L complex generated with HADDOCK 2.4 (Honorato et al. 2024), showing helix H3 inserted into the DNA major groove and the TAAT motif on the 5′‐3′ strand. The four residues under positive selection are highlighted (green and grey sticks). (F) Distinct spatial arrangements of the N‐IDRs of the HoxB4 proteins, revealing different orientations of Irx5 DNA (blue cartoon) in each complex.

To better understand the evolution of coding sequences in a structural and functional context, we modelled the protein structures and predicted the functional regions of *Xenopus tropicalis* HoxB4, and *X. laevis* HoxB4L and HoxB4S. All three proteins possess a conserved homeodomain and at least one intrinsically disordered region (IDR); however, they differ in the number, length, sequence composition, and structural arrangement of these IDRs (Figure 3A–C). Both *X. laevis* HoxB4S and *X. tropicalis* HoxB4 contain two IDRs – one located near the N‐terminus (N‐IDR) and another near the C‐terminus (C‐IDR). In contrast, *X. laevis* HoxB4L has undergone structural alterations: the C‐IDR is absent, and the N‐IDR is significantly shorter, comprising only 22 amino acid residues, compared with 90 residues in HoxB4S and 87 in *X. tropicalis* HoxB4. Sequence alignment of the N‐IDR regions revealed strong conservation within the 22‐residue segment corresponding to the HoxB4L N‐IDR (Figure 3D). Nonetheless, three of the four sites under positive selection in HoxB4L (Table S4) are located within the N‐IDR: glycine (G32), serine (S34), and phenylalanine (F35). These residues correspond to glutamine (Q), arginine (R), and glutamate (E) at positions 36, 38, and 39 in HoxB4S, and to positions 41, 43, and 44 in *X. tropicalis* HoxB4, respectively (Figure 3D). Notably, molecular docking simulations between HoxB4 proteins and *Irx5* gene segments (see Methods) revealed conservation in DNA binding by helix H3 (Figure 3E), despite the spatial arrangements of N‐IDRs correlate with distinct orientations of the *Irx5* DNA fragments in each complex (Figure 3F), leading to different docking results values. In fact, the modelling of the HoxB4L‐DNA complex yielded more favourable interaction metrics compared with the other two proteins, as indicated by HADDOCK scores (*X. laevis* HoxB4L = −203.7 ± 11.5; *X. laevis* HoxB4S = −112.8 ± 16.2; *X. tropicalis* HoxB4 = −178.0 ± 4.6) and RMSD values from the lowest‐energy structure (*X. laevis* HoxB4L = 1.1 ± 0.7; *X. laevis* HoxB4S = 4.8 ± 0.1; *X. tropicalis* HoxB4 = 3.0 ± 0.2) (see Methods). These findings indicate that HoxB4L is undergoing substantial structure divergence in IDRs, including changes in their number, length, and sequence. Given the role of IDRs in modulating DNA interactions and enabling regulatory flexibility, the structural divergence observed in HoxB4L likely reflects an adaptive trajectory towards functional innovation.

### 2.5 Combined Expression and Coding Sequence Divergence Is Specific to the *HoxB4* Pair

**Figure 4:**
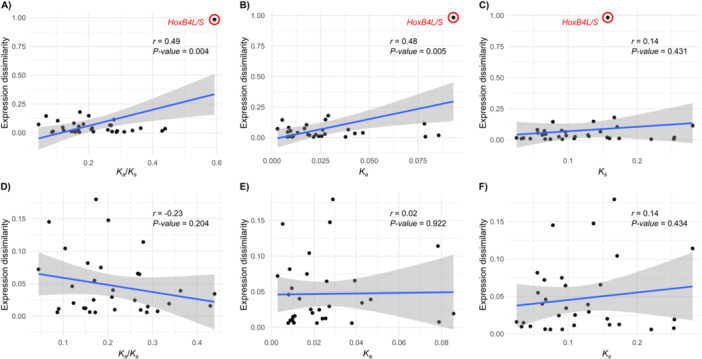
Correlation analyses between expression divergence and coding sequence evolution. Positive correlations are observed between expression dissimilarity (ED) and the ratio of nonsynonymous to synonymous substitutions (*K*_*a*_ */K*_*s*_) (A), nonsynonymous divergence (*K*_*a*_) (B), and synonymous divergence (*K*_*s*_) (C). However, these correlations are not significant when the outlier *HoxB4L/S* is excluded from the analyses (D–F).

The parallel divergence observed in both the expression and coding sequence of *HoxB4L* suggests an integrated evolutionary trajectory, probably shaped by selection for maternal neofunctionalization. Alternatively, this combined divergence may reflect a broader phenomenon, in which *Hox* homeologue pairs generally exhibit comparable rates of divergence in their regulatory elements and coding sequences – indicating a coupling between expression divergence and sequence evolution. To distinguish between these hypotheses, we calculated an index of divergence in expression patterns (named expression dissimilarity, ED) and tested whether it correlates with divergence parameters estimated for the coding sequence (*K*_*a*_, *K*_*s*_ and *K*_*a*_ */K*_*s*_) (Table S5). When all homeologue pairs were included in the analyses, ED was positively correlated with the rates of nonsynonymous (*K*_*a*_) and synonymous (*K*_*s*_) divergence, as well as with the ratio of nonsynonymous to synonymous divergence (*K*_*a*_ */K*_*s*_) (Figure 4A–C). However, the *HoxB4L/S* pair was a clear outlier, suggesting that this single point drives the positive correlations. Upon removing this pair from the analyses, all correlations lost significance (Figure 4D–F). Therefore, the combined changes in expression and coding sequences observed in *HoxB4L* do not represent a general feature of the *Hox* clusters, but rather reflect a unique set of molecular innovations specific to this homeologue. These results further support the hypothesis of an ongoing process of maternal neofunctionalization in *HoxB4L*, including both cis‐regulatory and protein structural changes.

## 3 Discussion

How does development evolve when it is governed by tightly regulated processes mediated by highly pleiotropic genes? Evo‐devo has emphasized the central role of regulatory evolution, particularly through the modulation of cis‐regulatory regions, which can fine‐tune gene function in specific spatiotemporal contexts (Carroll et al. 2004; Carroll 2005; Cañestro et al. 2007). In contrast, changes in the structure of transcription factors have traditionally been viewed as a minor source of developmental innovation. However, this perspective may underestimate their evolutionary potential (Hoekstra and Coyne 2007; Lynch and Wagner 2008; Wagner and Lynch 2008). The deleterious effects of mutations in multifunctional proteins can be mitigated by gene duplication, which introduces functional redundancy (Ohno 1970; Lynch and Conery 2000), or by the modular organization of protein domains, which can compartmentalize the effects of mutations to specific regions (Lynch and Wagner 2008; Cheatle Jarvela et al. 2014).

In this study, we investigated the evolutionary dynamics of duplicated *Hox* toolkit genes in the allotetraploid *Xenopus laevis*, which has retained nearly all copies since a WGD that occurred 17−18 Mya (Session et al. 2016; Kondo et al. 2017). Our findings show that most *Hox* homeologue pairs maintain highly similar expression profiles and average expression levels (Table S1), supporting a model of selection for gene dosage balance – where stoichiometric integrity in multisubunit complexes is preserved (Birchler and Veitia 2012). This mechanism likely underlies the remarkable retention of *Hox* duplicates following WGD. However, *HoxB4L* constitutes a notable exception, as it exhibits both maternal and zygotic expression (Figure 1), whereas *HoxB4S* and all other *Hox* genes in *X. laevis*, as well as *HoxB4* in *X. tropicalis*, are expressed exclusively from the zygotic genome during gastrulation (Wacker et al. 2004; Figure S1). The putative cis‐regulatory regions of *HoxB4L* are more divergent and evolve faster than those of *HoxB4S* or *X. laevis HoxB4* (Figure 2). These changes may have allowed their recognition by the maternal transcriptional machinery, thus contributing to its co‐option to maternally regulated stages. That said, given the interconnected nature of developmental regulatory networks, we cannot exclude co‐evolution of maternal transcription factors to accommodate these regulatory changes (Wagner and Lynch 2008; Pereira et al. 2022).

Coding sequences of *X. laevis Hox* homeologues predominantly evolve under strong purifying selection (median *K*_*a*_ */K*_*s*_ = 0.1788); however, *HoxB3L*, *HoxB4L*, *HoxB5L*, and *HoxB6L* are rapidly evolving (Table S3). To explore the mechanisms driving this acceleration, we tested two alternative hypotheses: (i) relaxation of selective constraints and (ii) adaptive evolution by positive selection (Lynch and Conery 2000). *HoxB3L* and *HoxB5L* did not show clear signatures of either process, suggesting a more complex evolutionary scenario. In contrast, *HoxB6L* shows strong evidence of relaxed selection (Table S4). This pattern aligns with expectations following gene duplication, where functional redundancy can reduce selective pressure, often leading to eventual gene loss (Ohno 1970; Lynch and Conery 2000). Indeed, *HoxB2L*, a gene from the same cluster, has undergone pseudogenization (Session et al. 2016; Kondo et al. 2017). The rapid evolution of *HoxB4L*, however, is explained by positive selection in at least four sites (Figure 3, Table S4). Notably, three of these sites are clustered within a disordered region near the N‐terminal portion of the protein (Figure 3). In addition to these adaptive substitutions, HoxB4L diverges from both its homeologue (*HoxB4S*) and an orthologue (*HoxB4* from *X. tropicalis*) in terms of the IDR architecture: this protein has lost C‐IDR and exhibits a shorter N‐IDR. These findings point to a modular evolution of the HoxB4L protein, involving changes in IDR number, sequence, and length. Given that IDRs are thought to mediate regulatory specificity in Hox proteins by facilitating interactions with diverse cofactors and signalling partners (Bondos and Hsiao 2012; Salomone et al. 2024), such modifications may bear significant evolutionary relevance. Their structural flexibility and reduced pleiotropic constraints, relative to conserved DNA‐binding domains, make IDRs particularly amenable to functional diversification. The observed changes in HoxB4L IDRs may therefore reflect a key route through which this protein acquired novel regulatory potential. This integrated pattern of structural and regulatory divergence does not extend to the entire *Hox* clusters, but instead appears to be a unique feature of the *HoxB4L/S* pair (Figure 4), specifically driven by molecular innovations in *HoxB4L*. Our findings strongly indicate that this gene is undergoing neofunctionalization to fulfil a new role during maternally regulated developmental stages.

In contrast to the widely recognized contribution of *Hox* cis‐regulatory and structural evolution to the vertebrate increase in complexity and diversification (Carroll 1995; Singh and Krumlauf 2022), the evolutionary implications of their recruitment into maternally regulated stages remain poorly understood. A notable example comes from the ant tribe Camponotini, where maternal expression of two *Hox* genes regulating early germline specification was essential in consolidating the obligate endosymbiotic association with *Blochmannia*, a key step in a major evolutionary transition in biological individuality (Rafiqi et al. 2020). The evolution of *bicoid* (*bcd*), the key anterior in *Drosophila*, represents a striking example of how gene duplication followed by regulatory and structural innovation can lead to major developmental roles. *Bcd* is maternally transcribed by nurse cells during oogenesis, and its mRNA localizes to the anterior pole of the oocyte (Johnston et al. 1989). Post‐fertilization, the translated protein forms an anteroposterior gradient critical for regulating downstream gene expression (Ochoa‐Espinosa et al. 2005). *Bcd* is a derived *Hox3* gene that underwent neofunctionalization after a tandem gene duplication in the ancestor of Cyclorrhaphan flies (Stauber et al. 1999). Non‐cyclorrhaphan flies retain *Hox3* genes with both maternal and zygotic expression, suggesting that, post‐duplication, one copy evolved as a maternal effect gene (*bcd*) while the other retained zygotic expression (*zerknullt*) (Stauber et al. 2002). The integration of *bcd* into early developmental gene regulatory networks was made possible through a combination of molecular innovations in *bcd* itself (Lynch and Desplan 2003; Onal et al. 2021) and in pre‐existing components of the regulatory system (Lemke et al. 2008; de Oliveira et al. 2017). It is conceivable that the recruitment of *HoxB4L* into a maternally regulated regulatory network in *X. laevis* may have involved structural modifications in maternal transcription factors and cis‐regulatory elements of downstream target genes. Future studies will help clarify how *HoxB4L* has become integrated into a maternally regulated developmental programme, and what evolutionary and developmental consequences this rewiring may entail.

## 4 Materials and Methods

### 4.1 Gene Expression Data and Analyses

RNA‐seq data were retrieved from Session et al. 2016, comprising TPM estimates for premature oocytes (oo12, oo34 and oo56), unfertilized eggs (egg), developmental stages (st08, st09, st10, st12, st15, st20, st25, st30, st35 and st40) and fourteen adult tissues (brain, eye, heart, intestine, kidney, liver, lung, muscle, ovary, pancreas, skin, spleen, stomach and testis). Because our analyses focused on temporal expression dynamics across development, expression values from adult tissues – primarily reflecting spatial variation – were averaged to generate a single adult expression estimate. Four of the 38 *Hox* homeologue pairs were excluded, either because one of the copies is a pseudogene (*HoxB2L*) (Kondo et al. 2017) or had undetectable expression (*HoxA2S*, *HoxC6S*, and *HoxA10L*). Expression data for the remaining 34 pairs were log_2_‐transformed (log_2_(TPM + 1)). Comparisons between homeologues included: (i) average expression levels via *T*‐tests; (ii) expression specificity using the *Tau* index (Yanai et al. 2005); and (iii) overall expression profiles using Pearson's correlation. Multiple testing correction was applied using the false discovery rate (*FDR*).

For comparative analyses in *X. tropicalis*, we used the high‐resolution temporal RNA‐seq dataset from Owens et al. (2016), which sampled embryos from two parallel, synchronously developing in vitro fertilizations (Clutch A and Clutch B). Eggs (time 0) and embryos were collected at 30‐min intervals during the first 24 h, followed by hourly sampling up to 66 h post‐fertilization. For Clutch A, polyA+ RNA libraries were sequenced across the entire 66‐h time series (up to approximately NF stage 42), and total RNA depleted of rRNA (rdRNA) was sequenced for the first 24 h. For Clutch B, only polyA+ RNA libraries were sequenced during the first 24 h, covering stages up to roughly NF 27. In addition, ribo‐seq data available on Xenbase (https://www.xenbase.org) were incorporated, providing expression information for early stages up to approximately NF 26. All gene expression analyses were conducted in R.

### 4.2 Cis‐Regulatory Region Analyses

Genomic regions (5Kb) upstream of *HoxB4L* and *HoxB4S* coding sequences from *X. laevis* (GCF_017654675.1) and *HoxB4* from another five amphibians – *Xenopus tropicalis* (GCF_000004195.4), *Nanorana parkeri* (GCF_000935625.1), *Geotrypetes seraphini* (GCF_902459505.1), *Rhinatrema bivittatum* (GCF_901001135.1), and *Microcaecilia unicolor* (GCF_901765095.1) were retrieved using the NCBI Genome Data Viewer (https://www.ncbi.nlm.nih.gov/gdv) (Rangwala et al. 2021) in July/2025. Sequences were aligned using MAFFT (Katoh 2002) within DAMBE v5.3.74 (Xia 2013), where substitution saturation was also assessed (Xia et al. 2003). The substitution saturation index was below critical values (ISS = 0.6019; ISS_c_ = 0.8108; *p* < 0.0001), indicating that these sequences are phylogenetically informative.

Tajima's relative rate test (Tajima 1993) was performed in MEGA v11 with *X. tropicalis* as outgroup (Tamura et al. 2021). Pairwise evolutionary distances (Tamura et al. 2021) were also estimated in MEGA.

To identify putative cis‐regulatory regions in *Xenopus laevis* and assess their evolutionary conservation, *X. laevis* sequences were excluded from the multispecies alignment to avoid circularity. Conservation scores (Siepel et al. 2005) were then calculated using PhastWeb (http://compgen.cshl.edu/phastweb/) (Ramani et al. 2019), based on the alignment of orthologous sequences from the five additional amphibian species described above. Both the sequence alignment and the corresponding phylogenetic tree were provided as input for the analysis. This tree was estimated by maximum likelihood using PhyML (Guindon et al. 2010) in the ATGC server (http://www.atgc-montpellier.fr/phyml/execution.php) after prediction of the best evolutionary model using the Smart Model Selection tool (Lefort et al. 2017) under the Akaike information criterion. Regions with *phastCons* conservation scores > 0.8 were designated putative cis‐regulatory elements and manually mapped back onto *X. laevis* and *X. tropicalis* sequences upstream of *HoxB4* homeologues/orthologues. We concatenated these putative cis‐regulatory motifs from *X. laevis* and *X. tropicalis*, and performed both Tajima's relative rate test (Tajima 1993) and estimation of pairwise evolutionary distances (Tamura et al. 2021) as above.

### 4.3 Coding Sequence Analyses

Transcripts of 37 pairs of *X. laevis Hox* homeologues and orthologues from the same five amphibians used in the cis‐regulatory region analyses were retrieved (October/2020 – March/2021) using NCBI Genome Data Viewer (https://www.ncbi.nlm.nih.gov/gdv) (Rangwala et al. 2021). *HoxB2* sequences were excluded due to pseudogenization of the *L* copy (Kondo et al. 2017). Open reading frames (ORFs) were predicted using NCBI ORFfinder (https://www.ncbi.nlm.nih.gov/orffinder/) (Hancock and Bishop 2004). Sequences from each paralogous group were codon‐aligned using MAFFT (Katoh 2002) in DAMBE 5.3.74 (Xia 2013), where substitution saturation was also assessed. The *HoxA3* alignment was removed from further analyses because substitution saturation is above critical values (Table S2) (Xia et al. 2003).

For each *Hox* gene alignment, Tajima's relative rate test, the selection of the best‐fitting evolutionary model, and the inference of maximum likelihood phylogenies were conducted as described above for the cis‐regulatory region analyses.

Branch‐site tests for relaxed and positive selection were conducted separately on each alignment, with the *L* and *S* copies each designated as the foreground/test branch in independent runs. To test for relaxation of selective strength, we used the RELAX method (Wertheim et al. 2015), implemented in the Datamonkey web server (https://www.datamonkey.org/) (Delport et al. 2010). This approach compares two models: (i) a null model in which the selection intensity parameter *k* is fixed to 1 across all branches, and (ii) an alternative model in which *k* is a free parameter. The parameter *k* acts as a scaling coefficient for the ω distribution along the test branches, allowing the detection of shifts in selective intensity across the evolutionary history of the focal group (here, each one the *X. laevis Hox* homeologues) while the reference branches correspond to *Hox* genes from five additional amphibian species (*X. tropicalis*, *N. parkeri*, *M. unicolor*, *G. seraphini*, and *R. bivittatum*). Model comparisons were performed using a likelihood ratio test (*LRT*). A significant *LRT* result indicates either intensified selection (*k* > 1), reflecting stronger purifying and/or positive selection on the test branches, or relaxed selection (*k* < 1), reflecting weaker selective constraints and a shift toward more neutral codon evolution relative to the reference branches (Wertheim et al. 2015). To test for positive selection, we employed two complementary methods: the adaptive Branch‐Site Random Effects Likelihood (aBSREL) test (Smith et al. 2015), implemented in DataMonkey (Delport et al. 2010), and the strict branch‐site test implemented in EasyCodeML (Yang 2007; Gao et al. 2019). Both approaches rely on the ratio of nonsynonymous to synonymous rates (*dN*/*dS* or *ω*), where *ω* ≈ 1 suggests neutral evolution, *ω* > 1 indicates positive selection, and *ω* < 1 indicates purifying selection. In both tests, two models are fitted: (i) a null model in which *ω* is constrained to be ≤ 1 across branches, and (ii) an alternative model that allows *ω* > 1 in the test/foreground branch. These models are compared using an *LRT*; a significant result provides evidence for positive selection. In EasyCodeML, significant *LRT*s are followed by a Bayes Empirical Bayes (BEB) to identify specific codons under positive selection (Yang 2005). All *p‐values* were adjusted for multiple tests using the false discovery rate (*FDR*) method.

### 4.4 Protein Structure Analyses

Coding sequences of *HoxB4L* and *HoxB4S* from *X. laevis*, along with the *X. tropicalis* orthologue, were translated and scanned for functional domains using InterPro (Blum et al. 2025). The resulting amino acid sequences were aligned using ClustalW (Thompson et al. 1994). Secondary structures were predicted with the PROTEUS2 server (Montgomerie et al. 2008). Three‐dimensional protein structures were modelled with the AlphaFold server (https://alphafoldserver.com/), powered by AlphaFold 3.0 (Abramson et al. 2024), using a seed value of 20. Model confidence was assessed using the predicted Local Distance Difference Test (pLDDT) metric, which provides per‐atom confidence scores on a 0−100 scale, with higher values indicating greater reliability (Abramson et al. 2024). For each model, the average of pLDDT was calculated across all Cα atoms.

To gain insights into the functional capabilities of the three proteins, we performed molecular docking analyses between their AlphaFold‐predicted tertiary structures and 18‐bp DNA fragments derived from the *Irx5* loci of *Xenopus laevis* (L and S homeologs) and *X. tropicalis*. *Irx5* is a known transcriptional target downstream of HoxB4 activity (Theokli et al. 2003). Each DNA fragment contained a conserved TAAT motif – characteristic of high‐affinity binding by homeodomain transcription factors, including Hox proteins (Svingen and Tonissen 2006) – flanked by 14 nucleotides located within the 5′ upstream regulatory region of each gene (see Figure S2). Three‐dimensional DNA structures were predicted using the AlphaFold server (Abramson et al. 2024). Docking was carried out using the Easy interface of HADDOCK 2.4 (https://rascar.science.uu.nl/haddock2.4/) (Honorato et al. 2024), a data‐driven docking server that predicts intermolecular interactions and ranks output clusters based on average scoring functions. For the docking of HoxB4L, the following amino acid residues were defined as active: R135, R137, R175, K178, Q182, N183, R185, K187, K189, K190 and K193. These sites are the DNA‐binding residues of the homeodomain (Piper et al. 1999). For the other two proteins, the corresponding residues were selected based on sequence alignment: for *X. laevis* HoxB4S – R143, R145, R183, K186, Q190, N191, R193, K195, K197, K198, and K201; and for *X. tropicalis* HoxB4 – R145, R147, R185, K188, Q192, N193, R195, K197, K199, K200, and K203. For all DNA fragments, the active interface was defined as the TAAT core motif, along with the two nucleotides immediately downstream (Figure S2), which are known to contribute to Hox‐specific recognition patterns (Svingen and Tonissen 2006). All other parameters were set to their default values. The resulting protein‐DNA complexes were evaluated based on three criteria: (i) the HADDOCK score, a weighted sum of van der Waals, electrostatic, desolvation, and restraint violation energies, where lower scores indicate better predicted interactions (Honorato et al. 2024); (ii) the RMSD relative to the lowest‐energy structure in each cluster, which estimates model convergence and structural similarities, with lower RMSD values indicating greater confidence (Honorato et al. 2024); and (iii) the spatial orientation of the helix H3 within the homeodomain, which typically inserts into the major groove of the DNA (Piper et al. 1999).

All structures were visualized and rendered using PyMOL Molecular Graphics System, Version 3.1 (Schrödinger LLC).

### 4.5 Expression and Coding Sequence Correlation Analysis

To assess whether divergence in expression and coding sequence in *HoxB4L* reflects a general trend in the *Hox* clusters, expression divergence (ED) was calculated as ED=1−|r|, where *r* is the Pearson correlation of expression profiles between homeologues. Coding sequence divergence was estimated as *K*_*a*_, *K*_*s*_, and *K*_*a*_ */K*_*s*_ ratios using EasyCodeml (Gao et al. 2019). Pearson correlation tests were performed in R to examine associations between ED and sequence divergence metrics.

## Conflicts of Interest

The authors declare no conflicts of interest.

## Supporting information

Supporting material revised.

## Data Availability

All gene expression and genome datasets used in this study were previously published and are publicly accessible. Full references and/or accession numbers are provided in the main text.
