# Supplementary material for: Escaping Constraints to Innovate: Maternal Neofunctionalization in a HoxB4 Duplicate
Source: J Exp Zool B Mol Dev Evol. 2026 Jan 12;346(2):141–51. doi: 10.1002/jezb.70012 (PMC12887915; doi:10.1002/jezb.70012)
Supplement: Supplementary file 1 — Supporting material revised. [file JEZ-346-141-s001.docx]

**Supplementary material**

**Tables**

**Table S1. Comparative gene expression analyses of *Hox* homeologues in *Xenopus laevis*.** Summary of expression analyses for 34 *Hox* homeologue pairs (*L* and *S* copies). Mean log₂(TPM + 1) values were compared using two-tailed *T*-tests. Tissue specificity was assessed using the *Tau* index; differences in *Tau* values between homeologue pairs are reported. Expression profiles were compared using Pearson’s correlation (*r*). All *P-values* were adjusted for multiple testing using *FDR* correction.

| **Gene** | ***T*-test** | | ***Tau* index** | | **Pearson’s correlation** | |
| --- | --- | --- | --- | --- | --- | --- |
|  | Mean | *FDR* | Individual^1^ | Difference^2^ | *r* | *FDR* |
| ***HoxA1L*** | 2.00 | 0.68 | 0.71 | -0.08 | 0.82 | <0.0002 |
| ***HoxA1S*** | 0.88 |  | 0.79 |  |  |  |
| ***HoxA3L*** | 1.46 | 0.62 | 0.71 | 0.07 | 0.99 | <10 ^-16^ |
| ***HoxA3S*** | 1.79 |  | 0.78 |  |  |  |
| ***HoxA4L*** | 0.50 | 0.96 | 0.74 | 0.07 | 0.93 | <10^-6^ |
| ***HoxA4S*** | 0.53 |  | 0.67 |  |  |  |
| ***HoxA5L*** | 0.66 | 0.96 | 0.75 | -0.05 | 0.95 | <10^-6^ |
| ***HoxA5S*** | 0.73 |  | 0.80 |  |  |  |
| ***HoxA6L*** | 0.42 | 0.96 | 0.82 | 0.02 | 0.94 | <10^-6^ |
| ***HoxA6S*** | 0.37 |  | 0.80 |  |  |  |
| ***HoxA7L*** | 1.95 | 0.89 | 0.75 | -0.08 | 0.98 | <10^-9^ |
| ***HoxA7S*** | 1.10 |  | 0.83 |  |  |  |
| ***HoxA9L*** | 1.73 | 0.96 | 0.70 | 0.03 | 0.96 | <10^-7^ |
| ***HoxA9S*** | 1.47 |  | 0.67 |  |  |  |
| ***HoxA11L*** | 0.83 | 0.96 | 0.71 | -0.02 | 0.97 | <10^-9^ |
| ***HoxA11S*** | 0.52 |  | 0.73 |  |  |  |
| ***HoxA13L*** | 0.33 | 0.68 | 0.83 | -0.01 | 0.96 | <10^-7^ |
| ***HoxA13S*** | 0.13 |  | 0.85 |  |  |  |
| ***HoxB1L*** | 0.77 | 0.89 | 0.82 | 0.04 | 0.85 | <10^-4^ |
| ***HoxB1S*** | 1.45 |  | 0.77 |  |  |  |
| ***HoxB3L*** | 1.65 | 0.96 | 0.62 | -0.01 | 0.93 | <10^-6^ |
| ***HoxB3S*** | 2.10 |  | 0.62 |  |  |  |
| ***HoxB4L*** | 2.98 | 0.68 | 0.36 | -0.31 | **0.02** | **0.954** |
| ***HoxB4S*** | 1.92 |  | 0.67 |  |  |  |
| ***HoxB5L*** | 0.76 | 0.9 | 0.70 | 0.00 | 0.98 | <10^-9^ |
| ***HoxB5S*** | 1.67 |  | 0.70 |  |  |  |
| ***HoxB6L*** | 1.49 | 0.96 | 0.78 | 0.07 | 0.89 | <10^-4^ |
| ***HoxB6S*** | 1.87 |  | 0.71 |  |  |  |
| ***HoxB7L*** | 2.09 | 0.96 | 0.71 | 0.02 | 0.99 | <10^-11^ |
| ***HoxB7S*** | 1.42 |  | 0.69 |  |  |  |
| ***HoxB8L*** | 0.16 | 0.38 | 0.89 | 0.15 | 0.85 | <10^-4^ |
| ***HoxB8S*** | 1.95 |  | 0.74 |  |  |  |
| ***HoxB9L*** | 1.13 | 0.89 | 0.75 | 0.03 | 0.97 | <10^-8^ |
| ***HoxB9S*** | 2.11 |  | 0.72 |  |  |  |
| ***HoxC3L*** | 1.34 | 0.96 | 0.73 | -0.03 | 0.99 | <10^-12^ |
| ***HoxC3S*** | 1.13 |  | 0.76 |  |  |  |
| ***HoxC4L*** | 1.23 | 0.96 | 0.76 | -0.03 | 0.99 | <10^-10^ |
| ***HoxC4S*** | 0.87 |  | 0.79 |  |  |  |
| ***HoxC5L*** | 1.09 | 0.96 | 0.74 | -0.03 | 0.99 | <10^-13^ |
| ***HoxC5S*** | 1.12 |  | 0.77 |  |  |  |
| ***HoxC8L*** | 1.65 | 0.96 | 0.74 | 0.01 | 0.99 | <10^-13^ |
| ***HoxC8S*** | 1.59 |  | 0.74 |  |  |  |
| ***HoxC9L*** | 0.69 | 0.96 | 0.81 | 0.09 | 0.95 | <10^-7^ |
| ***HoxC9S*** | 0.72 |  | 0.72 |  |  |  |
| ***HoxC10L*** | 1.07 | 0.96 | 0.73 | -0.04 | 0.99 | <10^-11^ |
| ***HoxC10S*** | 1.03 |  | 0.77 |  |  |  |
| ***HoxC11L*** | 0.63 | 0.96 | 0.79 | 0.01 | 0.99 | <10^-11^ |
| ***HoxC11S*** | 0.77 |  | 0.77 |  |  |  |
| ***HoxC12L*** | 1.02 | 0.96 | 0.72 | 0.00 | 0.97 | <10^-8^ |
| ***HoxC12S*** | 1.20 |  | 0.73 |  |  |  |
| ***HoxC13L*** | 0.95 | 0.96 | 0.69 | 0.03 | 0.93 | <10^-6^ |
| ***HoxC13S*** | 0.78 |  | 0.66 |  |  |  |
| ***HoxD1L*** | 2.09 | 0.96 | 0.78 | 0.02 | 0.99 | <10^-13^ |
| ***HoxD1S*** | 2.21 |  | 0.76 |  |  |  |
| ***HoxD3L*** | 0.98 | 0.96 | 0.75 | -0.03 | 0.99 | <10^-11^ |
| ***HoxD3S*** | 0.89 |  | 0.79 |  |  |  |
| ***HoxD4L*** | 1.09 | 0.68 | 0.83 | -0.04 | 0.98 | <10^-10^ |
| ***HoxD4S*** | 0.31 |  | 0.87 |  |  |  |
| ***HoxD8L*** | 0.96 | 0.89 | 0.66 | -0.08 | 0.98 | <10^-9^ |
| ***HoxD8S*** | 0.56 |  | 0.74 |  |  |  |
| ***HoxD9L*** | 0.89 | 0.96 | 0.77 | 0.02 | 0.99 | <10^-13^ |
| ***HoxD9S*** | 0.72 |  | 0.75 |  |  |  |
| ***HoxD10L*** | 0.74 | 0.96 | 0.75 | 0.01 | 0.99 | <10^-11^ |
| ***HoxD10S*** | 0.71 |  | 0.74 |  |  |  |
| ***HoxD11L*** | 0.44 | 0.96 | 0.81 | -0.01 | 0.92 | <10^-5^ |
| ***HoxD11S*** | 0.29 |  | 0.82 |  |  |  |
| ***HoxD13L*** | 0.81 | 0.96 | 0.69 | -0.02 | 0.90 | <10^-5^ |
| ***HoxD13S*** | 0.79 |  | 0.71 |  |  |  |

^1^ Raw *Tau* index values calculated for each homeologue.

^2^ Absolute difference in *Tau* values between *L* and *S* copies.

**Table S2. Assessment of sequence substitution saturation in *Hox* sequences.** Results of substitution saturation tests for 36 *Hox* genes based on codon-aligned sequences of *X. laevis* homeologues and 5 amphibian orthologues. The index of substitution saturation (ISS) was calculated and compared with the critical value for a symmetrical tree (ISS_c_). All *P-values* were adjusted for multiple testing using *FDR* correction. Genes with ISS < ISS_c_ and *FDR* < 0.05 are considered phylogenetically informative and were retained for downstream analyses. *HoxA3* exceeds the critical threshold and was excluded.

| **Gene** | **ISS** | **ISS_c_ symmetrical** | ***FDR*** | **Phylogenetically**  **informative?** |
| --- | --- | --- | --- | --- |
| ***HoxA1*** | 0.5401 | 0.7879 | 0 | TRUE |
| ***HoxA2*** | 0.1981 | 0.7925 | 0 | TRUE |
| ***HoxA3*** | **0.9373** | **0.7985** | **0** | **FALSE** |
| ***HoxA4*** | 0.5379 | 0.7824 | 0 | TRUE |
| ***HoxA5*** | 0.4033 | 0.7786 | 0 | TRUE |
| ***HoxA6*** | 0.5043 | 0.7696 | 0 | TRUE |
| ***HoxA7*** | 0.3378 | 0.7675 | 0 | TRUE |
| ***HoxA9*** | 0.4668 | 0.7746 | 0 | TRUE |
| ***HoxA10*** | 0.2896 | 0.7924 | 0 | TRUE |
| ***HoxA11*** | 0.2056 | 0.7814 | 0 | TRUE |
| ***HoxA13*** | 0.2363 | 0.7804 | 0 | TRUE |
| ***HoxB1*** | 0.6157 | 0.7836 | 0 | TRUE |
| ***HoxB3*** | 0.6382 | 0.7973 | 0 | TRUE |
| ***HoxB4*** | 0.4604 | 0.7728 | 0 | TRUE |
| ***HoxB5*** | 0.4692 | 0.7798 | 0 | TRUE |
| ***HoxB6*** | 0.2683 | 0.7666 | 0 | TRUE |
| ***HoxB7*** | 0.5212 | 0.7664 | 0 | TRUE |
| ***HoxB8*** | 0.1794 | 0.7708 | 0 | TRUE |
| ***HoxB9*** | 0.5021 | 0.7783 | 0 | TRUE |
| ***HoxC3*** | 0.4506 | 0.7969 | 0 | TRUE |
| ***HoxC4*** | 0.5102 | 0.7904 | 0 | TRUE |
| ***HoxC5*** | 0.2071 | 0.769 | 0 | TRUE |
| ***HoxC6*** | 0.5371 | 0.7706 | 0 | TRUE |
| ***HoxC8*** | 0.2671 | 0.7736 | 0 | TRUE |
| ***HoxC9*** | 0.6242 | 0.7759 | 0 | TRUE |
| ***HoxC10*** | 0.2437 | 0.7912 | 0 | TRUE |
| ***HoxC11*** | 0.2167 | 0.7835 | 0 | TRUE |
| ***HoxC12*** | 0.2771 | 0.7766 | 0 | TRUE |
| ***HoxC13*** | 0.6388 | 0.783 | 0 | TRUE |
| ***HoxD1*** | 0.4573 | 0.7846 | 0 | TRUE |
| ***HoxD3*** | 0.2235 | 0.7971 | 0 | TRUE |
| ***HoxD4*** | 0.3135 | 0.77 | 0 | TRUE |
| ***HoxD8*** | 0.3588 | 0.7723 | 0 | TRUE |
| ***HoxD9*** | 0.3243 | 0.7791 | 0 | TRUE |
| ***HoxD10*** | 0.6097 | 0.7913 | 0 | TRUE |
| ***HoxD11*** | 0.4464 | 0.7776 | 0 | TRUE |
| ***HoxD13*** | 0.3628 | 0.7828 | 0 | TRUE |

**Table S3. Results of Tajima’s relative rate tests comparing *L* and *S* *Hox* homeologues in *Xenopus laevis*.** Tajima’s relative rate test was performed for each *Hox* gene using *Xenopus tropicalis* as an outgroup. The number of identical and divergent sites among the three sequences (*X. laevis L*, *X. laevis S*, and *X. tropicalis*) is shown, along with the number of unique differences in each. The χ² statistic tests for differences in substitution rates between the *L* and *S* homeologues. *P-values* were adjusted for multiple comparisons using the false discovery rate (*FDR*) method. Significant results (*FDR* < 0.05) indicate unequal evolutionary rates between homeologues.

| **Gene** | **Identical sites (all three)** | **Divergent sites (all three)** | **Unique differences  (*L*)** | **Unique differences  (*S*)** | **Unique differences  *X. tropicalis*** | ***χ2*** | ***FDR*** |
| --- | --- | --- | --- | --- | --- | --- | --- |
| ***HoxA1*** | 880 | 3 | 30 | 21 | 29 | 1.59 | 0.415 |
| ***HoxA2*** | 1046 | 4 | 18 | 19 | 17 | 0.03 | 0.956 |
| ***HoxA4*** | 704 | 0 | 6 | 18 | 16 | 6.00 | 0.074 |
| ***HoxA5*** | 788 | 0 | 5 | 11 | 12 | 2.25 | 0.321 |
| ***HoxA6*** | 650 | 0 | 15 | 13 | 24 | 0.14 | 0.907 |
| ***HoxA7*** | 566 | 3 | 10 | 16 | 20 | 1.38 | 0.453 |
| ***HoxA9*** | 686 | 7 | 20 | 21 | 40 | 0.02 | 0.956 |
| ***HoxA10*** | 947 | 7 | 21 | 26 | 52 | 0.53 | 0.699 |
| ***HoxA11*** | 832 | 1 | 11 | 21 | 20 | 3.13 | 0.308 |
| ***HoxA13*** | 821 | 1 | 10 | 10 | 43 | 0.00 | 1.000 |
| ***HoxB1*** | 802 | 2 | 26 | 17 | 62 | 1.88 | 0.382 |
| ***HoxB3*** | 1010 | 3 | 46 | 16 | 62 | 14.52 | **0.002** |
| ***HoxB4*** | 502 | 4 | 41 | 14 | 42 | 13.25 | **0.002** |
| ***HoxB5*** | 553 | 7 | 55 | 15 | 54 | 22.86 | **0.000** |
| ***HoxB6*** | 521 | 7 | 54 | 7 | 41 | 36.21 | **0.000** |
| ***HoxB7*** | 572 | 1 | 20 | 11 | 38 | 2.61 | 0.327 |
| ***HoxB8*** | 666 | 1 | 7 | 6 | 46 | 0.08 | 0.925 |
| ***HoxB9*** | 546 | 4 | 7 | 21 | 79 | 7.00 | 0.059 |
| ***HoxC3*** | 962 | 12 | 41 | 67 | 55 | 6.26 | 0.074 |
| ***HoxC4*** | 852 | 1 | 3 | 10 | 22 | 3.77 | 0.732 |
| ***HoxC5*** | 788 | 0 | 5 | 11 | 12 | 2.25 | 0.321 |
| ***HoxC6*** | 678 | 1 | 3 | 7 | 13 | 1.60 | 0.415 |
| ***HoxC8*** | 678 | 2 | 5 | 9 | 32 | 1.14 | 0.489 |
| ***HoxC9*** | 238 | 0 | 2 | 7 | 53 | 2.78 | 0.321 |
| ***HoxC10*** | 822 | 10 | 19 | 30 | 169 | 2.47 | 0.321 |
| ***HoxC11*** | 883 | 1 | 7 | 8 | 28 | 0.07 | 0.925 |
| ***HoxC12*** | 703 | 5 | 17 | 17 | 47 | 0.00 | 1.000 |
| ***HoxC13*** | 827 | 1 | 4 | 10 | 49 | 2.57 | 0.321 |
| ***HoxD1*** | 779 | 1 | 29 | 34 | 39 | 0.40 | 0.732 |
| ***HoxD3*** | 1117 | 2 | 28 | 37 | 52 | 1.25 | 0.476 |
| ***HoxD4*** | 673 | 1 | 4 | 5 | 28 | 0.11 | 0.917 |
| ***HoxD8*** | 635 | 2 | 11 | 11 | 28 | 0.00 | 1.000 |
| ***HoxD9*** | 743 | 1 | 9 | 11 | 43 | 0.20 | 0.873 |
| ***HoxD10*** | 978 | 0 | 11 | 3 | 19 | 4.57 | 0.146 |
| ***HoxD11*** | 778 | 0 | 9 | 6 | 29 | 0.60 | 0.686 |
| ***HoxD13*** | 778 | 1 | 23 | 18 | 32 | 0.61 | 0.686 |

**Table S4. Codon-based selection analyses for *Xenopus* laevis *Hox* homeologues.** Results of three codon-based tests for selection: RELAX (*k* values and *FDR*), aBSREL (likelihood ratio test and *FDR*), and the branch-site model in EasyCodeML (likelihood ratio test, *FDR*, and sites identified under positive selection using Bayes Empirical Bayes, BEB). Values are shown separately for each homeologue (*L* and *S*) of each *Hox* gene. In the RELAX test, *k* < 1 indicates relaxation and *k* > 1 indicates intensification of selection. The BEB column lists codons under positive selection (posterior probability > 0.95), with positions indicated both in the alignment and in the HoxB4L protein (in parentheses).

| **Gene** | **RELAX** | | **aBSREL** | | **EasyCodeML** | | |
| --- | --- | --- | --- | --- | --- | --- | --- |
|  | ***k*** | ***FDR*** | ***LRT*** | ***FDR*** | ***LRT*** | ***FDR*** | **BEB** |
| ***HoxA1L*** | 0.97 | 1.0 | 0.0 | 1.0 | 1.0 | 1.0 |  |
| ***HoxA1S*** | 0.13 | 0.18 | 0.01 | 1.0 | 0.87 | 1.0 |  |
| ***HoxA2L*** | 1.36 | 1.0 | 0.0 | 1.0 | 1.0 | 1.0 |  |
| ***HoxA2S*** | 0.28 | 0.31 | 6.19 | 0.23 | 0.11 | 0.2 |  |
| ***HoxA4L*** | 0.9 | 1.0 | 0.0 | 1.0 | 1.0 | 1.0 |  |
| ***HoxA4S*** | 1.17 | 1.0 | 0.0 | 1.0 | 1.0 | 1.0 |  |
| ***HoxA5L*** | 0.99 | 1.0 | 0.0 | 1.0 | 1.0 | 1.0 |  |
| ***HoxA5S*** | 0.63 | 0.51 | 0.0 | 1.0 | 0.88 | 1.0 |  |
| ***HoxA6L*** | 0.55 | 0.51 | 0.0 | 1.0 | 1.0 | 1.0 |  |
| ***HoxA6S*** | 0.36 | 0.31 | 0.0 | 1.0 | 0.98 | 1.0 |  |
| ***HoxA7L*** | 0.0 | 0.26 | 0.43 | 1.0 | 0.57 | 1.0 |  |
| ***HoxA7S*** | 1.44 | 0.83 | 0.0 | 1.0 | 1.0 | 1.0 |  |
| ***HoxA9L*** | 0.00 | 0.18 | 0.0 | 1.0 | 0.94 | 1.0 |  |
| ***HoxA9S*** | 0.00 | **0.0** | 5.80 | 0.23 | 0.08 | 0.72 |  |
| ***HoxA10L*** | 13.80 | 0.07 | 8.96 | 0.08 | 0.01 | 0.2 |  |
| ***HoxA10S*** | 1.38 | 0.83 | 0.0 | 1.0 | 0.96 | 1.0 |  |
| ***HoxA11L*** | 1.22 | 0.98 | 0.0 | 1.0 | 1.0 | 1.0 |  |
| ***HoxA11S*** | 0.48 | 0.18 | 0.0 | 1.0 | 0.92 | 1.0 |  |
| ***HoxA13L*** | 0.51 | 0.85 | 0.0 | 1.0 | 1.0 | 1.0 |  |
| ***HoxA13S*** | 0.46 | 0.29 | 0.0 | 1.0 | 0.96 | 1.0 |  |
| ***HoxB1L*** | 50.0 | 0.18 | 0.0 | 1.0 | 1.0 | 1.0 |  |
| ***HoxB1S*** | 18.8 | 0.18 | 0.0 | 1.0 | 1.0 | 1.0 |  |
| ***HoxB3L*** | 0.52 | 0.31 | 8.52 | 0.08 | 0.01 | 0.2 |  |
| ***HoxB3S*** | 0.98 | 1.0 | 0.0 | 1.0 | 1.0 | 1.0 |  |
| ***HoxB4L*** | 0.82 | 0.79 | 19.39 | **0.0** | 0.0001 | **0.01** | 14 (14) E 0.830,21 (21) Q 0.875,23 (23) F 0.901,29 (29) S 0.950*,**41 (32) G 1.000****,42 (33) Q 0.777,**43 (34) S 0.999****,**44 (35) F 0.996****,62 (53) N 0.712,63 (54) N 0.589,71 (62) L 0.825,72 (63) R 0.687,76 (67) H 0.652,**91 (76) T 0.996****,125 (104) Y 0.624,131 (110) H 0.660,135 (114) L 0.901,149 (128) D 0.846,162 (139) S 0.911,182 (159) S 0.885,221 (198) M 0.871,226 (203) H 0.691 |
| ***HoxB4S*** | 0.36 | 0.31 | 0.00 | 1.0 | 0.97 | 1.0 |  |
| ***HoxB5L*** | 0.72 | 0.26 | 2.96 | 0.55 | 0.002 | 0.07 |  |
| ***HoxB5S*** | 0.80 | 0.98 | 0.0 | 1.0 | 1.0 | 1.0 |  |
| ***HoxB6L*** | 0.40 | **0.0** | 0.0 | 1.0 | 0.53 | 1.0 |  |
| ***HoxB6S*** | 1.48 | 0.83 | 0.0 | 1.0 | 1.00 | 1.0 |  |
| ***HoxB7L*** | 0.64 | 0.44 | 0.0 | 1.0 | 0.95 | 1.0 |  |
| ***HoxB7S*** | 0.59 | 0.44 | 0.1 | 1.0 | 1.0 | 1.0 |  |
| ***HoxB8L*** | 0.32 | 0.44 | 0.0 | 1.0 | 1.0 | 1.0 |  |
| ***HoxB8S*** | 21.99 | 0.79 | 0.0 | 1.0 | 1.0 | 1.0 |  |
| ***HoxB9L*** | 1.11 | 1.0 | 0.0 | 1.0 | 0.13 | 1.0 |  |
| ***HoxB9S*** | 0.19 | 0.26 | 3.87 | 0.42 | 1.0 | 1.0 |  |
| ***HoxC3L*** | 0.00 | 0.67 | 2.33 | 1.0 | 1.0 | 1.0 |  |
| ***HoxC3S*** | 0.51 | 0.32 | 0.0 | 1.0 | 0.6 | 1.0 |  |
| ***HoxC4L*** | 3.94 | 1.0 | 0.0 | 1.0 | 1.0 | 1.0 |  |
| ***HoxC4S*** | 25.54 | 0.26 | 0.0 | 1.0 | 1.0 | 1.0 |  |
| ***HoxC5L*** | 0.92 | 1.0 | 0.0 | 1.0 | 1.0 | 1.0 |  |
| ***HoxC5S*** | 0.38 | 0.23 | 0.0 | 1.0 | 1.0 | 1.0 |  |
| ***HoxC6L*** | 0.96 | 1.0 | 0.0 | 1.0 | 1.0 | 1.0 |  |
| ***HoxC6S*** | 0.37 | 0.32 | 0.0 | 1.0 | 0.79 | 1.0 |  |
| ***HoxC8L*** | 0.52 | 0.32 | 0.0 | 1.0 | 1.0 | 1.0 |  |
| ***HoxC8S*** | 0.63 | 0.43 | 0.0 | 1.0 | 1.0 | 1.0 |  |
| ***HoxC9L*** | 0.93 | 1.0 | 0.0 | 1.0 | 1.0 | 1.0 |  |
| ***HoxC9S*** | 0.19 | 0.32 | 0.0 | 1.0 | 1.0 | 1.0 |  |
| ***HoxC10L*** | 1.38 | 0.98 | 0.0 | 1.0 | 1.0 | 1.0 |  |
| ***HoxC10S*** | 1.40 | 0.83 | 0.0 | 1.0 | 1.0 | 1.0 |  |
| ***HoxC11L*** | 0.98 | 1.0 | 0.0 | 1.0 | 1.0 | 1.0 |  |
| ***HoxC11S*** | 0.01 | 1.0 | 0.06 | 1.0 | 0.5 | 1.0 |  |
| ***HoxC12L*** | 0.66 | 0.51 | 0.0 | 1.0 | 1.0 | 1.0 |  |
| ***HoxC12S*** | 0.49 | 0.28 | 0.0 | 1.0 | 0.52 | 1.0 |  |
| ***HoxC13L*** | 0.16 | 0.51 | 0.01 | 1.0 | 0.77 | 1.0 |  |
| ***HoxC13S*** | 48.44 | 0.07 | 0.0 | 1.0 | 1.0 | 1.0 |  |
| ***HoxD1L*** | 5.13 | 0.43 | 0.0 | 1.0 | 0.66 | 1.0 |  |
| ***HoxD1S*** | 1.06 | 1.0 | 0.0 | 1.0 | 0.97 | 1.0 |  |
| ***HoxD3L*** | 0.03 | 0.07 | 0.0 | 1.0 | 0.2 | 1.0 |  |
| ***HoxD3S*** | 1.00 | 1.0 | 0.66 | 1.0 | 0.21 | 1.0 |  |
| ***HoxD4L*** | 0.32 | 0.26 | 0.0 | 1.0 | 0.87 | 1.0 |  |
| ***HoxD4S*** | 0.73 | 0.83 | 5.39 | 0.25 | 0.99 | 1.0 |  |
| ***HoxD8L*** | 1.03 | 1.0 | 0.0 | 1.0 | 1.0 | 1.0 |  |
| ***HoxD8S*** | 0.27 | 0.26 | 0.0 | 1.0 | 1.0 | 1.0 |  |
| ***HoxD9L*** | 50.00 | 0.08 | 0.0 | 1.0 | 1.0 | 1.0 |  |
| ***HoxD9S*** | 1.04 | 1.0 | 0.0 | 1.0 | 1.0 | 1.0 |  |
| ***HoxD10L*** | 1.98 | 0.39 | 3.37 | 0.49 | 0.02 | 0.28 |  |
| ***HoxD10S*** | 0.21 | 1.0 | 0.0 | 1.0 | 1.0 | 1.0 |  |
| ***HoxD11L*** | 1.56 | 0.69 | 9.00 | 0.08 | 1.0 | 1.0 |  |
| ***HoxD11S*** | 42.11 | 0.85 | 4.28 | 0.38 | 1.0 | 1.0 |  |
| ***HoxD13L*** | 0.95 | 1.0 | 0.0 | 1.0 | 1.0 | 1.0 |  |
| ***HoxD13S*** | 1.24 | 0.85 | 0.0 | 1.0 | 1.0 | 1.0 |  |

**Table S5. Expression and coding sequence divergence between *Hox* homeologue pairs in *Xenopus laevis*.** Expression divergence was calculated as 1 - |*r*|, where *r* is the Pearson correlation coefficient between the expression profiles of homeologue pairs. Sequence divergence includes *K_a_/K_s_*, and absolute *K_a_* and *K_s_* values. Missing values (-) in the expression column indicate a lack of detectable expression in one homeologue; missing sequence values reflect substitution saturation, precluding reliable estimates.

| **Gene** | | **Expression** | **Sequence** | | |
| --- | --- | --- | --- | --- | --- |
|  |  | **Dissimilarity** | ***K_a_/K_s_*** | ***K_a_*** | ***K_s_*** |
| ***HoxA1*** | 0.180 | | 0.173 | 0.029 | 0.167 |
| ***HoxA2*** | - | | 0.126 | 0.016 | 0.127 |
| ***HoxA3*** | 0.002 | | - | - | - |
| ***HoxA4*** | 0.074 | | 0.184 | 0.017 | 0.091 |
| ***HoxA5*** | 0.054 | | 0.170 | 0.010 | 0.056 |
| ***HoxA6*** | 0.065 | | 0.270 | 0.026 | 0.096 |
| ***HoxA7*** | 0.020 | | 0.123 | 0.019 | 0.157 |
| ***HoxA9*** | 0.040 | | 0.363 | 0.047 | 0.129 |
| ***HoxA10*** | - | | 0.167 | 0.025 | 0.148 |
| ***HoxA11*** | 0.025 | | 0.168 | 0.019 | 0.110 |
| ***HoxA13*** | 0.040 | | 0.212 | 0.013 | 0.063 |
| ***HoxB1*** | 0.148 | | 0.201 | 0.028 | 0.137 |
| ***HoxB3*** | 0.066 | | 0.267 | 0.039 | 0.147 |
| ***HoxB4*** | 0.984 | | 0.592 | 0.093 | 0.157 |
| ***HoxB5*** | 0.019 | | 0.337 | 0.086 | 0.253 |
| ***HoxB6*** | 0.114 | | 0.280 | 0.078 | 0.280 |
| ***HoxB7*** | 0.012 | | 0.150 | 0.024 | 0.160 |
| ***HoxB8*** | 0.145 | | 0.068 | 0.005 | 0.078 |
| ***HoxB9*** | 0.034 | | 0.439 | 0.043 | 0.097 |
| ***HoxC3*** | 0.008 | | 0.312 | 0.079 | 0.253 |
| ***HoxC4*** | 0.015 | | 0.290 | 0.010 | 0.035 |
| ***HoxC5*** | 0.006 | | 0.287 | 0.021 | 0.074 |
| ***HoxC6*** | - | | 0.162 | 0.007 | 0.044 |
| ***HoxC8*** | 0.006 | | 0.160 | 0.011 | 0.067 |
| ***HoxC9*** | 0.046 | | 0.120 | 0.008 | 0.068 |
| ***HoxC10*** | 0.011 | | 0.090 | 0.010 | 0.109 |
| ***HoxC11*** | 0.010 | | 0.215 | 0.010 | 0.045 |
| ***HoxC12*** | 0.030 | | 0.209 | 0.028 | 0.130 |
| ***HoxC13*** | 0.072 | | 0.045 | 0.003 | 0.066 |
| ***HoxD1*** | 0.006 | | 0.172 | 0.038 | 0.220 |
| ***HoxD3*** | 0.013 | | 0.146 | 0.026 | 0.174 |
| ***HoxD4*** | 0.016 | | 0.430 | 0.011 | 0.026 |
| ***HoxD8*** | 0.024 | | 0.260 | 0.023 | 0.087 |
| ***HoxD9*** | 0.006 | | 0.087 | 0.008 | 0.092 |
| ***HoxD10*** | 0.010 | | 0.274 | 0.009 | 0.033 |
| ***HoxD11*** | 0.082 | | 0.155 | 0.009 | 0.055 |
| ***HoxD13*** | 0.104 | | 0.104 | 0.178 | 0.171 |

**Figures**

**
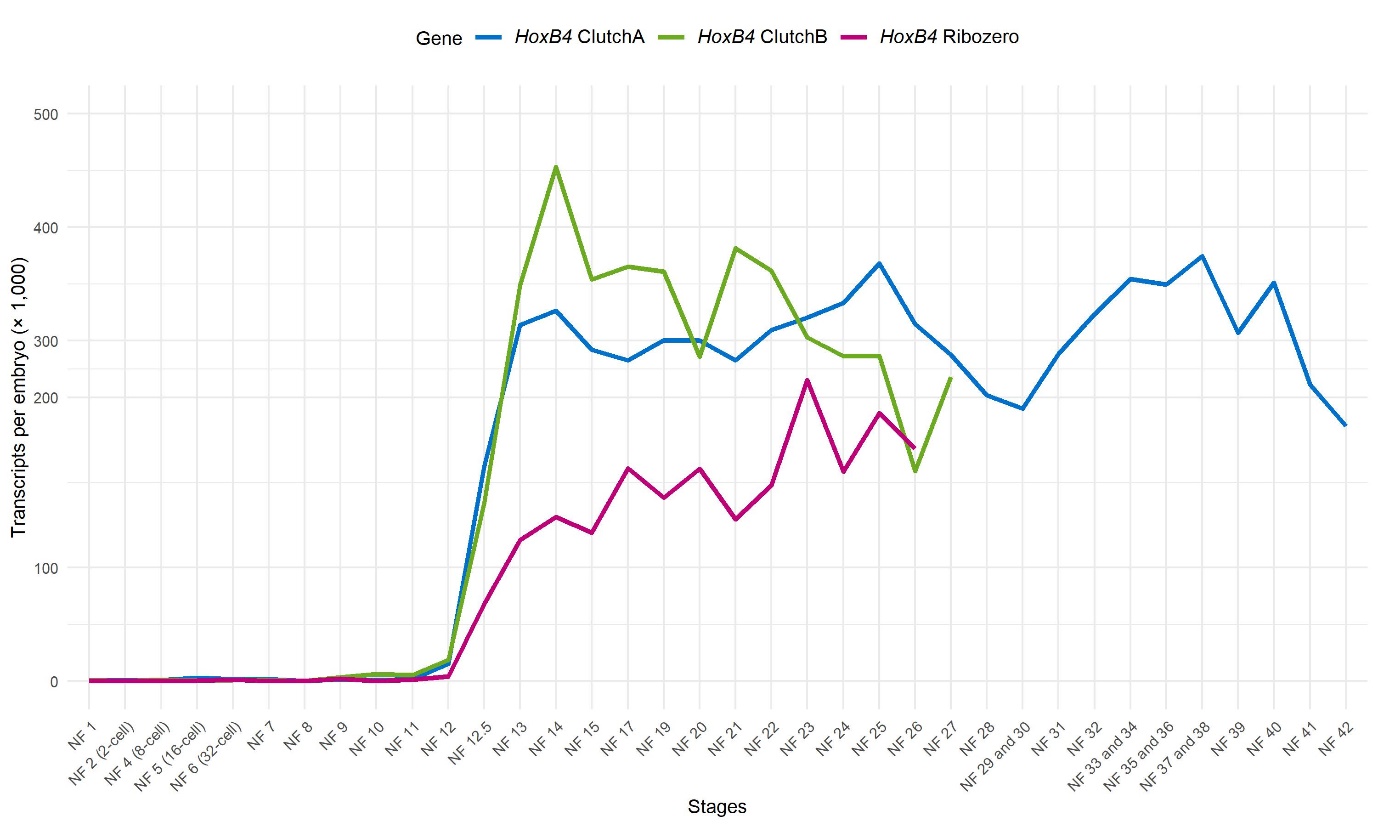
**

**Figure S1. Temporal expression profile of *HoxB4* in *Xenopus tropicalis*.** Expression levels of *HoxB4* across embryonic development are shown based on RNA-seq data from Owens et al. (2016). Profiles are shown for two independent, synchronously developing in vitro fertilization clutches (Clutch A, blue; Clutch B, green) and for ribo-depleted RNA-seq data (Ribo-zero, magenta). The x-axis indicates developmental stages, and the y-axis represents transcript abundance expressed as transcripts per embryo (×1,000). No detectable *HoxB4* expression is observed during maternally regulated stages (NF 1-8/9), with expression initiating after the maternal-to-zygotic transition and increasing during gastrulation and subsequent developmental stages.

**Figure S2**. **Nucleotide sequences of *Irx5* genes used to model DNA fragments for molecular docking analyses with HoxB4 proteins.** The 5′ upstream regions (lowercase) and the first coding sequences (uppercase) of each gene are shown. The 18-bp segments containing the TAAT motif (bold) are highlighted and the two immediately downstream nucleotides, defined as the active sites for the docking simulations, are underlined. All sequences were retrieved from the Alliance of Genome Resources using the “Genomic with full introns ±500 bp upstream/downstream” option.
